# Examining the Impact of COVID-19 on Loneliness and Social Isolation Among Affordable Housing Residents

**DOI:** 10.1093/geroni/igab046.3596

**Published:** 2021-12-17

**Authors:** Marilyn Mock, Roisin Goebelbecker, Sherry Pomerantz, Jennifer DeGennaro, Elyse Perweiler

**Affiliations:** 1 Fair Share Support Services, Inc./Fair Share Housing Development, Camden, New Jersey, United States; 2 Rowan School of Osteopathic Medicine, Stratford, New Jersey, United States

## Abstract

Loneliness and social isolation are serious public health concerns associated with higher risks of clinical depression, suicidal ideation, coronary artery disease, stroke, functional decline, an increased risk of developing dementia and cancer mortality. Recent reports indicate the prevalence and dangers of loneliness and social isolation have increased as a result of the COVID-19 pandemic, especially among older populations. In order to address these concerns among residents living at Northgate II (NGII), a 302-unit affordable housing development in Camden, NJ, Fair Share Support Services, Inc. (FSSS), the non-profit arm of Fair Share Housing Development, collaborated with the New Jersey Institute for Successful Aging (NJISA) and the DHHS-funded Geriatric Workforce Enhancement Program (GWEP) to develop a loneliness/social isolation survey using two evidenced-based tools, the UCLA Loneliness Scale and the Steptoe Social Isolation Index. FSSS piloted the loneliness and social isolation survey with 192 low-income minority older adults residing at NGII. Results indicate that 49% of the NGII residents surveyed fall into 5 "at-risk" categories: 1) lonely and isolated (9%), 2) lonely/somewhat isolated (8%), 3 ) lonely/not isolated (9%), 4) isolated/somewhat lonely (9%), and 5) isolated/not lonely (14%). FSSS, will utilize survey results and follow-up interviews to tailor social service/other interventions to meet the needs and preferences of residents with the goal of preventing serious health problems associated with loneliness and social isolation, allowing residents to age in place.

